# Supplementary material for: Incidence, characteristics, and risk factors of new liver disorders 3.5 years post COVID-19 pandemic in the Montefiore Health System in Bronx
Source: PLoS One. 2024 Jun 13;19(6):e0303151. doi: 10.1371/journal.pone.0303151 (PMC11175509; doi:10.1371/journal.pone.0303151)
Supplement: S1 Table — (DOCX) [file pone.0303151.s001.docx]

**Supplemental Table 1.** LD and OMOP concept ids

| **Abnormal liver function tests (LFTs)** | |
| --- | --- |
| 4042563 | Liver enzymes abnormal |
| 438878 | Liver function tests abnormal |
| 45769139 | Elevated liver enzymes level |
| **Steatosis** | |
| 4059290 | Steatosis of liver |
| **Unspecified LD** | |
| 194984 | Disease of liver |
| 194990 | Inflammatory disease of liver |
| **Advanced liver failure** | |
| 4058695 | Toxic liver disease with fibrosis and cirrhosis of liver |
| 4064161 | Cirrhosis of liver |
| 4245975 | Hepatic failure |
| 4340390 | Chronic hepatic failure |
| **Alcoholic liver** | |
| 193256 | Alcoholic fatty liver |
| 196463 | Alcoholic cirrhosis |
| 201612 | Alcoholic liver damage |
| 4340385 | Alcoholic fibrosis and sclerosis of liver |
| 4340386 | Alcoholic hepatic failure |
| 46269816 | Ascites due to alcoholic cirrhosis |
| 46269818 | Hepatic coma due to alcoholic liver failure |
| **Biliary cirrhosis** | |
| 192675 | Biliary cirrhosis |
| **Other** | |
| 4267417 | Hepatic fibrosis |
| 4340394 | Hepatic sclerosis |
| 4340948 | Hepatic fibrosis with hepatic sclerosis |
