# Supplementary material for: Incidence, characteristics, and risk factors of new liver disorders 3.5 years post COVID-19 pandemic in the Montefiore Health System in Bronx
Source: PLoS One. 2024 Jun 13;19(6):e0303151. doi: 10.1371/journal.pone.0303151 (PMC11175509; doi:10.1371/journal.pone.0303151)
Supplement: S2 Table — (DOCX) [file pone.0303151.s002.docx]

**Supplemental Table 2.** LRTI and OMOP concept ids

| **Concept ID** | **Condition** | **Count** |
| --- | --- | --- |
| 8689 | Influenza due to unidentified influenza virus with other respiratory manifestations | 211 |
| 920135 | Acute bronchiolitis due to human metapneumovirus | 9 |
| 35207931 | Influenza due to other identified influenza virus with other respiratory manifestations | 325 |
| 35207933 | Respiratory syncytial virus pneumonia | 21 |
| 35207934 | Parainfluenza virus pneumonia | 1 |
| 35207935 | Human metapneumovirus pneumonia | 7 |
| 35207937 | Viral pneumonia, unspecified | 17 |
| 35207938 | Pneumonia due to Streptococcus pneumoniae | 12 |
| 35207939 | Pneumonia due to Hemophilus influenzae | 3 |
| 35207940 | Pneumonia due to Klebsiella pneumoniae | 4 |
| 35207941 | Pneumonia due to Pseudomonas | 1 |
| 35207942 | Pneumonia due to streptococcus, group B | 1 |
| 35207943 | Pneumonia due to other streptococci | 3 |
| 35207944 | Pneumonia due to Escherichia coli | 15 |
| 35207945 | Pneumonia due to other Gram-negative bacteria | 1 |
| 35207947 | Pneumonia due to other specified bacteria | 2 |
| 35207948 | Unspecified bacterial pneumonia | 4 |
| 35207949 | Chlamydial pneumonia | 2 |
| 35207950 | Pneumonia due to other specified infectious organisms | 9 |
| 35207951 | Pneumonia in diseases classified elsewhere | 4452 |
| 35207952 | Bronchopneumonia, unspecified organism | 1 |
| 35207953 | Lobar pneumonia, unspecified organism | 14 |
| 35207956 | Pneumonia, unspecified organism | 6 |
| 35207957 | Acute bronchitis due to Mycoplasma pneumoniae | 7 |
| 35207962 | Acute bronchitis due to respiratory syncytial virus | 14 |
| 35207963 | Acute bronchitis due to rhinovirus | 353 |
| 35207965 | Acute bronchitis due to other specified organisms | 13 |
| 35207966 | Acute bronchitis, unspecified | 493 |
| 35207967 | Acute bronchiolitis due to respiratory syncytial virus | 1 |
| 35207968 | Acute bronchiolitis due to other specified organisms | 4 |
| 35207969 | Acute bronchiolitis, unspecified | 2 |
| 45533545 | Pneumonia due to Methicillin susceptible Staphylococcus aureus | 8 |
| 45557620 | Pneumonia due to Methicillin resistant Staphylococcus aureus | 5 |
| 45581841 | Influenza due to identified novel influenza A virus with other manifestations | 211 |
| 45586661 | Influenza due to unidentified influenza virus with unspecified type of pneumonia | 9 |
| 45596276 | Influenza due to identified novel influenza A virus with pneumonia | 325 |
