# Supplementary material for: Incidence, characteristics, and risk factors of new liver disorders 3.5 years post COVID-19 pandemic in the Montefiore Health System in Bronx
Source: PLoS One. 2024 Jun 13;19(6):e0303151. doi: 10.1371/journal.pone.0303151 (PMC11175509; doi:10.1371/journal.pone.0303151)
Supplement: S3 Table — Respondents answered yes or no to each question. (DOCX) [file pone.0303151.s003.docx]

**Supplemental Table 3.** Social determinant of health questionnaires. Respondents answered yes or no to each question.

1. Worried about money for food
2. Worried about housing quality
3. Worried about housing situation
4. Lack of health care transportation
5. Health care cost burden
6. Utilities shut threat
7. Need legal help
8. Need child/adult care
