# Supplementary material for: Incidence, characteristics, and risk factors of new liver disorders 3.5 years post COVID-19 pandemic in the Montefiore Health System in Bronx
Source: PLoS One. 2024 Jun 13;19(6):e0303151. doi: 10.1371/journal.pone.0303151 (PMC11175509; doi:10.1371/journal.pone.0303151)
Supplement: S4 Table — These percentages are not mutually exclusive and do add up more than 100% (i.e., patients could have multiple disorders). There were no statistical significances between groups. (DOCX) [file pone.0303151.s004.docx]

**Supplemental Table 4.** Distribution of incident LD. These percentages are not mutually exclusive and do add up more than 100% (i.e., patients could have multiple disorders). There were no statistical significances between groups.

| **Conditions** | | **COVID-19 (n=557)** | **Hospitalized COVID-19 (n=262)** | **Non-COVID (n=532)** | **Hospitalized LRTI**  **(n=69)** |
| --- | --- | --- | --- | --- | --- |
| **Abnormal LFTs (Total)** | | 226 (40.57%) | 109 (41.60%) | 198 (37.22%) | 28 (40.58%) |
| 4042563 | Liver enzymes abnormal | 23 (4.13%) | 12 (4.58%) | 27 (5.08%) | 4 (5.80%) |
| 438878 | Liver function tests abnormal | 35 (6.28%) | 15 (5.73%) | 32 (6.02%) | 7 (10.14%) |
| 45769139 | Elevated liver enzymes level | 168 (30.16%) | 82 (31.30%) | 139 (26.13%) | 17 (24.64%) |
| **Steatosis** | |  |  |  |  |
| 4059290 | Steatosis of liver | 179 (32.14%) | 78 (29.77%) | 191 (35.90%) | 20 (28.99%) |
| **Unspecified LD (Total)** | | 115 (20.65%) | 53 (20.23%) | 126 (23.68%) | 15 (21.74%) |
| 194984 | Disease of liver | 101 (18.13%) | 45 (17.18%) | 113 (21.24%) | 12 (17.39%) |
| 194990 | Inflammatory disease of liver | 14 (2.51%) | 8 (3.05%) | 13 (2.44%) | 3 (4.35%) |
| **Advanced liver failure (Total)** | | 54 (9.69%) | 33 (12.60%) | 31 (5.83%) | 8 (11.59%) |
| 4064161 | Cirrhosis of liver | 43 (7.72%) | 25 (9.54%) | 26 (4.89%) | 7 (10.14%) |
| 4245975 | Hepatic failure | 8 (1.44%) | 5 (1.91%) | 5 (0.94%) | 1 (1.45%) |
| 4340390 | Chronic hepatic failure | 3 (0.54%) | 3 (1.15%) | 0 (0.00%) | 0 (0.00%) |
| **Alcoholic** **liver diseases (Total)** | | 18 (3.23%) | 14 (5.34%) | 14 (2.63%) | 4 (5.80%) |
| 193256 | Alcoholic fatty liver | 1 (0.18%) | 1 (0.38%) | 1 (0.19%) | 1 (1.45%) |
| 196463 | Alcoholic cirrhosis | 10 (1.80%) | 6 (2.29%) | 6 (1.13%) | 2 (2.90%) |
| 201612 | Alcoholic liver damage | 1 (0.18%) | 1 (0.38%) | 1 (0.19%) | 1 (1.45%) |
| 4340385 | Alcoholic fibrosis and sclerosis of liver | 1 (0.18%) | 1 (0.38%) | 0 (0.00%) | 0 (0.00%) |
| 46269816 | Ascites due to alcoholic cirrhosis | 5 (0.90%) | 5 (1.91%) | 6 (1.13%) | 0 (0.00%) |
| **Biliary** **cirrhosis** | |  |  |  |  |
| 192675 | Biliary cirrhosis | 0 (0.00%) | 0 (0.00%) | 1 (0.19%) | 0 (0.00%) |
| **Other** |  |  |  |  |  |
| 4267417 | Hepatic fibrosis | 6 (1.08%) | 3 (1.15%) | 3 (0.56%) | 0 (0.00%) |
